# Diagnostic and prognostic value of T‐wave amplitude difference between supine and orthostatic electrocardiogram in children and adolescents with postural orthostatic tachycardia syndrome

**DOI:** 10.1111/anec.12747

**Published:** 2020-02-29

**Authors:** Yuwen Wang, Yi Xu, Fang Li, Ping Lin, Juan Zhang, Runmei Zou, Cheng Wang

**Affiliations:** ^1^ Department of Pediatric Cardiovasology The Children's Medical Center The Second Xiangya Hospital Central South University Changsha China; ^2^ Institute of Pediatrics Central South University Changsha China

**Keywords:** adolescents, children, electrocardiography, orthostatic position, postural orthostatic tachycardia syndrome, supine position

## Abstract

**Objective:**

To investigate the diagnostic and prognostic value of T‐wave amplitude difference between supine and orthostatic electrocardiogram (ECG) in children and adolescents with postural orthostatic tachycardia syndrome (POTS).

**Methods:**

A total of 100 children and adolescents (POTS group, 50 males and 50 females, aged at 11.0 ± 2.4 years) diagnosed as POTS were enrolled from August 2013 to July 2016. Seventy‐one children were matched as the control group according to age and sex. All cases completed the supine and orthostatic ECG.

**Results:**

(a) Compared with the control group, the T‐wave amplitude difference in leads I, II, aVL, V_4_, V_5,_ and V_6_ and the heart rate (HR) difference increased in POTS group. (b) Logistic regression analysis: The T‐wave amplitude difference in leads V_4_, V_5_, and V_6_ and HR difference have statistical significance for POTS diagnosis. (c) Diagnostic test evaluation: When HR difference was ≥ 15 times/min, T‐wave amplitude difference in lead V_5_ was ≥0.15 mV, T‐wave amplitude difference in leads V_4_ and V_6_ were ≥0.10 mV, and the sensitivity and specificity of POTS diagnosis were 35.0% and 88.7%. (d) Follow‐up: There was no significant difference in HR difference and T‐wave amplitude difference in the nonresponse groups. In the response group, the T‐wave amplitude difference in lead V_4_ was reduced than the initial value.

**Conclusions:**

The HR difference and T‐wave amplitude difference in leads V4, V5, and V6 between supine and orthostatic ECG are of help in assisting the diagnosis of POTS but no obviously significance on prognosis estimation of it.

## 1 INTRODUCTION

Postural orthostatic tachycardia syndrome (POTS) is the presence of orthostatic intolerance (OI) symptoms, and the patient's heart rate (HR) increases by ≥40 times/min in the upright test or head‐up tilt test (HUTT) within 10 min compared to the supine position and (or) the maximum heart rate reaches the standard (≥130 times/min at 6 ~ 12 years old; ≥125 times/min at 13 ~ 18 years old); the simultaneous reduction of systolic blood pressure <20 mmHg, diastolic blood pressure drop <10 mmHg, and except other significant diseases that affect the cardiovascular system or the autonomic nervous system (Sheldon et al., 2015; Wang et al., 2018). The prevalence of POTS is approximately 0.2%. Most patients present with POTS between the ages of 15 ~ 25 years, and more than 75% are women (Sheldon et al., 2015). Chen et al. (2011 reported that female accounted for 53.5% of Chinese children and adolescents with POTS. The main clinical manifestations are OI symptoms, such as dizziness, palpitation, fatigue, blurred vision, chest tightness, and amaurosis. Severe cases may have syncope attacks (Kizilbash et al., 2014; Mizumaki, 2011; Stewart, 2002). POTS is a chronic systemic disease, which can significantly impairs patients' physical functioning, social functioning, and other aspects significantly reduce patients' quality of life (Agarwal, Garg, & Sarkar, 2007; Bagai et al., 2011; Benrud‐Larson et al., 2002; Sousa, Lebreiro, Freitas, & Maciel, 2012).

At present, POTS is diagnosed based on clinical manifestations combined with HUTT (Wang et al., 2018). However, HUTT has certain risks, such as temporary aphasia (Chu, Wang, et al., 2014), arrhythmia (Prabhu, Pillai, & Shenthar, 2017), and convulsions (Wang et al., 2013), which can cause psychological fear of subjects, especially those who are positive for HUTT (Chu, Wu, et al., 2014). Therefore, it is necessary to find a more convenient and safe method on POTS diagnosis. The main pathogenesis of POTS is central hypovolemia after standing up (Sheldon et al., 2015), which can lead to autonomic nervous dysfunction and affect ECG waveform changes. ECG has been widely used in clinic diagnosis. It has the advantages of convenient operation, high security, and low price. Ran and Wang (2014) reported T wave and ST segment on supine and orthostatic ECG were meaningful for evaluating autonomic nervous function, but there is lack of research on the clinic diagnosis value on supine and orthostatic ECG for POTS in children and adolescent. In this research, we are trying to investigate the diagnostic and prognostic value of T‐wave amplitudes changes between supine and orthostatic ECG in children and adolescents with POTS.

## 2 SUBJECTS AND METHOD

### 2.1 Study subjects

Totally, 100 cases of children and adolescents (50 males and 50 females, with an average age of 11.0 ± 2.4 years), with unexplained syncope or presyncope symptoms, diagnosed as POTS by HUTT were enrolled from Pediatric Specialist Clinic of the Second Xiangya Hospital of Central South University from August 2013 to July 2016. A total of 71 children and adolescents (42 males and 29 females, with an average age of 10.3 ± 2.6 years) were selected as the control group. The subjects were examined in detail for medical history, physical examination, and tests as blood biochemistry (fasting blood glucose and myocardial enzyme), 12‐lead ECG, 24‐hr dynamic ECG, chest X‐ray, echocardiogram, head CT or MRI images, and no abnormalities were found. Exclusion of organic cardiopulmonary, neurogenic, and psychogenic diseases on all cases. HUTT was approved after the guardians signed the informed consent. HUTT is a noninvasive examination which has been approved by the Ethics Committee of the Second Xiangya Hospital of Central South University.

### 2.2 Methods

#### 2.2.1 Head‐up tilt test

Subjects should stop using all drugs that affect autonomic function for more than 5 half‐lives before examination and discontinue diets that may affect autonomic function (Wang et al., 2018). Fasting and drinking prohibition should be maintained at least 4 hr before the test. To avoid distracting the subjects, the test environment should be quiet, dim and at adaptation temperature. Subjects and their guardians should be informed of the announcements and the possible risks before the test, and the guardians of subjects should sign a written informed consent. All subjects underwent HUTT from 8:00 a.m. to 11:00 a.m. at room temperature (20 ~ 24)°C. Subjects rested in the supine position for 10 min and emptied the bladder. The tilting device is SHUT‐100 tilt test monitoring software system of Beijing Standley Technology Co., LTD. Subjects were lying on an inclined diagnostic bed with ankle and knee bands fixed to avoid flexion. During the examination, the blood pressure and the 12‐lead ECG were monitored and recorded. The subjects lay quietly for 10 min, and the basic state HR, blood pressure and 12‐lead ECG were recorded. Within 15 s, the patients were converted to 60° with the head height and foot low tilt position. The HR, blood pressure and ECG were continuously monitored and recorded until the test was terminated after a positive reaction, and the supine position was restored within 10 s.

#### 2.2.2 Electrocardiogram

Before ECG recording, five half‐lives of cardiovascular active drugs and drugs affecting autonomic nervous function were stopped. The ECG was done on the day before HUTT. Children were asked to maintain in the supine position, and the 12‐lead ECG was recorded by SR‐1000A ECG comprehensive automatic analyzer from Zhongshan in Guangdong Province of China. The children were also asked to stand, with the electrode in the original position, until the HR was stable, and the 12‐lead ECG was recorded in orthostatic synchronization. No filtering device was used for sampling, and each sampling point was 2 ms. After routine collection of stable waveform for 30 s, the classification number was stored in the computer to establish case files. Gain at 1 mV = 10 mm, and paper speed at 25 mm/s. Using the initial position of Q wave as the reference level for the T‐wave amplitude measurement, the positive T‐wave amplitude was perpendicular to the peak of waveform from the reference horizontal line edge, the negative T‐wave amplitude was perpendicular from the lower edge of the reference horizontal line to the bottom of the waveform, and the two‐phase T‐wave amplitude was the algebraic sum of positive phase amplitude and negative phase amplitude. The three clear cardiac cycles of sinus rhythm were measured and averaged. The HR difference was HR in the standing position minus the HR in the supine position, and the T‐wave amplitude difference in 12 leads was the amplitude on supine ECG minus the value in the standing.

#### 2.2.3 Treatment

Health education was taken in the 100 children and adolescents with POTS, including psychological guidance of children and their families, avoidance of syncope inducement, drinking more water, keeping urine clear (urination twice in the morning and afternoon), assuring enough sleep time (>8 hr/day), erect training (standing against the wall, 2 times/day, 30 min/times), oral rehydration salts Ⅲ (made in Xi 'an Anjian Pharmaceutical Co., LTD, approval number: H20090205) 5.125 g/bag (each bag was divided into 250 ml water for oral administration, 2 bags/day; halve the dose for children under 6 years old) and oral metoprolol (from Astrazeneca Pharmaceutical Co., LTD, production batch number: 326201), 0.5 ~ 1.0 mg/kg, oral, 2 times/day.

#### 2.2.4 Follow‐up results

Forty children and adolescents with POTS were completely followed up. The follow‐up period ranged from 3 weeks to 1 year, with a median of 1.33 months (1.00, 4.65). During the follow‐up, the medical history was inquired, and HUTT, 12‐lead ECG in the supine and orthostatic position were reexamined. The treatment effect: Reexamining HUTT for 10 min, if the HR increased by <40 times/min and the maximum HR does not reach the standard of corresponding age group was considered as having a response to the treatment; otherwise, it was considered as having no response to the treatment.

### 2.3 Statistical analysis

SPSS 22.0 statistical software was used for all data analyses, the measurement data were presented as mean ± standard deviation (Mean ± *SD*), and *t* test was used for comparison between groups. Logistic regression analysis was used for multivariate analysis. The receiver operating characteristic (ROC) curve was used to evaluate the sensitivity and specificity of predictive indicators for judge the predictive effect. The area under the curve (AUC) was used to express the predictive ability of predictive indicators. When the Youden index (the sum of sensitivity and specificity and then minus 1) is the largest, its sensitivity and specificity reach the best, and this node is selected as the boundary value of the prediction index. *p < .*05 was considered as having statistically significant. The Kaplan–Meier curve was used to show the trend of cumulative response rate of patients over time.

## 3 RESULTS

### 3.1 Comparison of HR and the 12‐lead T‐wave amplitudes between supine and orthostatic ECG

**Figure 1:**
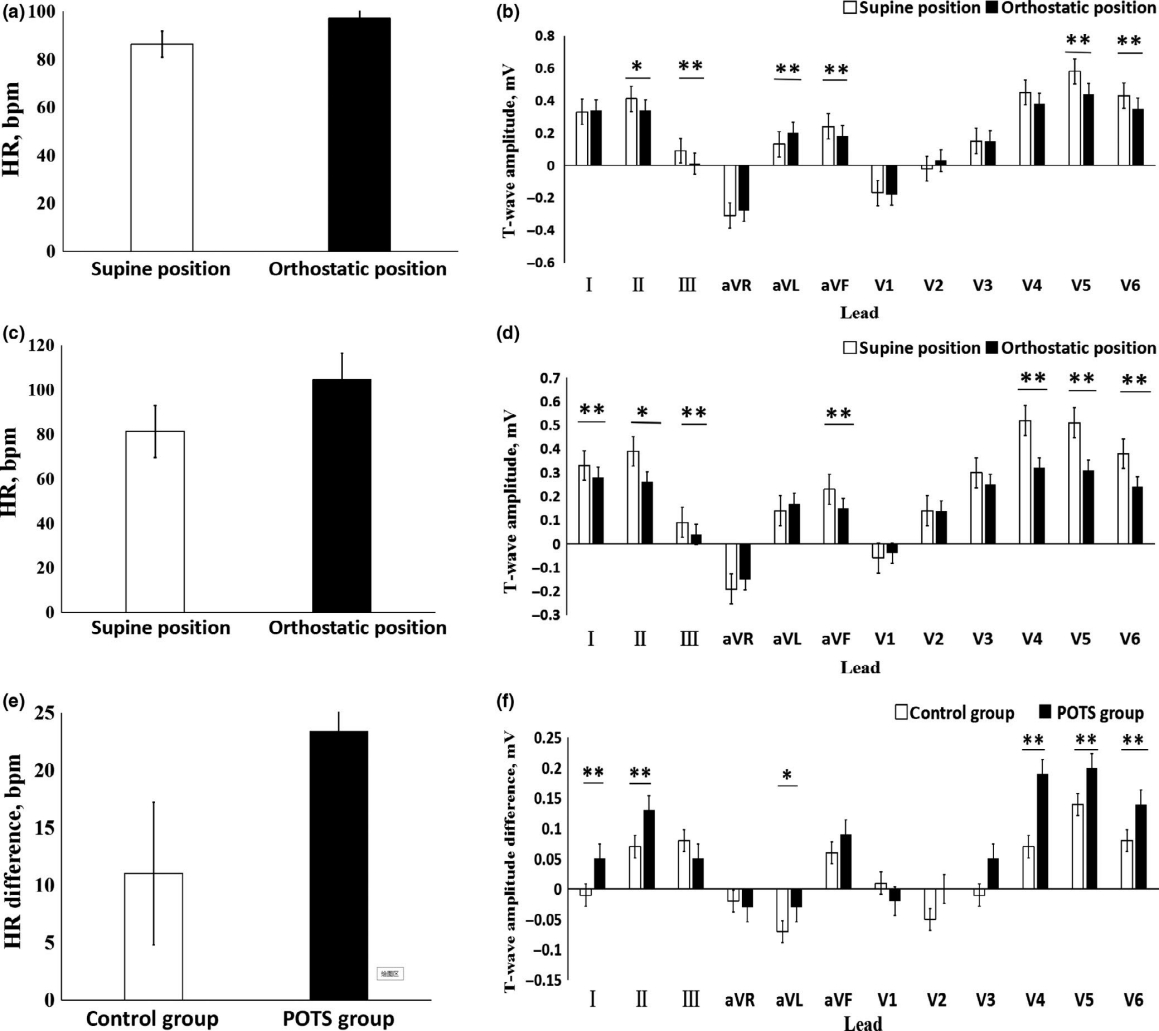
The comparison of HR and T‐wave amplitude in 12 leads between supine and orthostatic electrocardiogram (ECG). (a) The HR in the supine and orthostatic ECG of the control group. (b) The T‐wave amplitude in the supine and orthostatic ECG of the control group. (c) The HR in the supine and orthostatic ECG of POTS group. (d) The T‐wave amplitude in the supine and orthostatic ECG of POTS group. (e) The difference of HR in the supine and orthostatic ECG between POTS group and control group. (f) The difference of T‐wave amplitude in the supine and orthostatic ECG between POTS group and control group. * *p* < .05, ** *p* < .01

In control group, the HR increased (*p* < .01) and the T‐wave amplitude in leads Ⅱ, Ⅲ, aVF, V_5_, and V_6_ decreased (*p* < .05), while the T‐wave amplitude in lead aVL increased (*p* < .05) on the orthostatic ECG than on the supine ECG. In POTS group, the HR increased (*p* < .01), while the T‐wave amplitude in leads Ⅰ, Ⅱ, Ⅲ, aVF, V_4_, V_5_, and V_6_ decreased (*p* < .05) on the orthostatic ECG than on the supine ECG. The HR difference was increased (*p* < .01) and the T‐wave amplitude difference in leads Ⅰ, Ⅱ, aVL, V_4_, V_5_, and V_6_ also increased (*p* < .05) in POTS group compared with control group (Figure 1).

### 3.2 Logistic regression analysis

Logistic regression analysis was conducted on the HR difference and T‐wave amplitude difference in leads I, II, V_4_, V_5,_ and V_6_ between POTS group and control group. It was found that HR differences and T‐wave amplitude difference in leads V_4_, V_5,_ and V_6_ had statistical significance for POTS diagnosis (*p* < .05) (Table 1).

**Table 1:** Logistic regression analysis of HR difference and T‐wave amplitude difference in leads I, II, V_4_, V_5,_ and V_6_

		Regression Coefficient	*SE*	Wald	*p* value	Dominance Ratio (OR)
HR difference		−0.064	0.016	15.368	.000	0.938
T‐wave amplitude difference	Ⅰ	3.361	2.166	2.408	.121	28.825
	Ⅱ	−0.982	1.862	0.278	.598	0.375
	V_4_	3.349	1.280	6.844	.009	28.483
	V_5_	−7.274	2.827	6.621	.010	0.001
	V_6_	10.242	3.972	6.649	.010	28,053.121

### 3.3 Draw receiver operating characteristic curve

**Figure 2:**
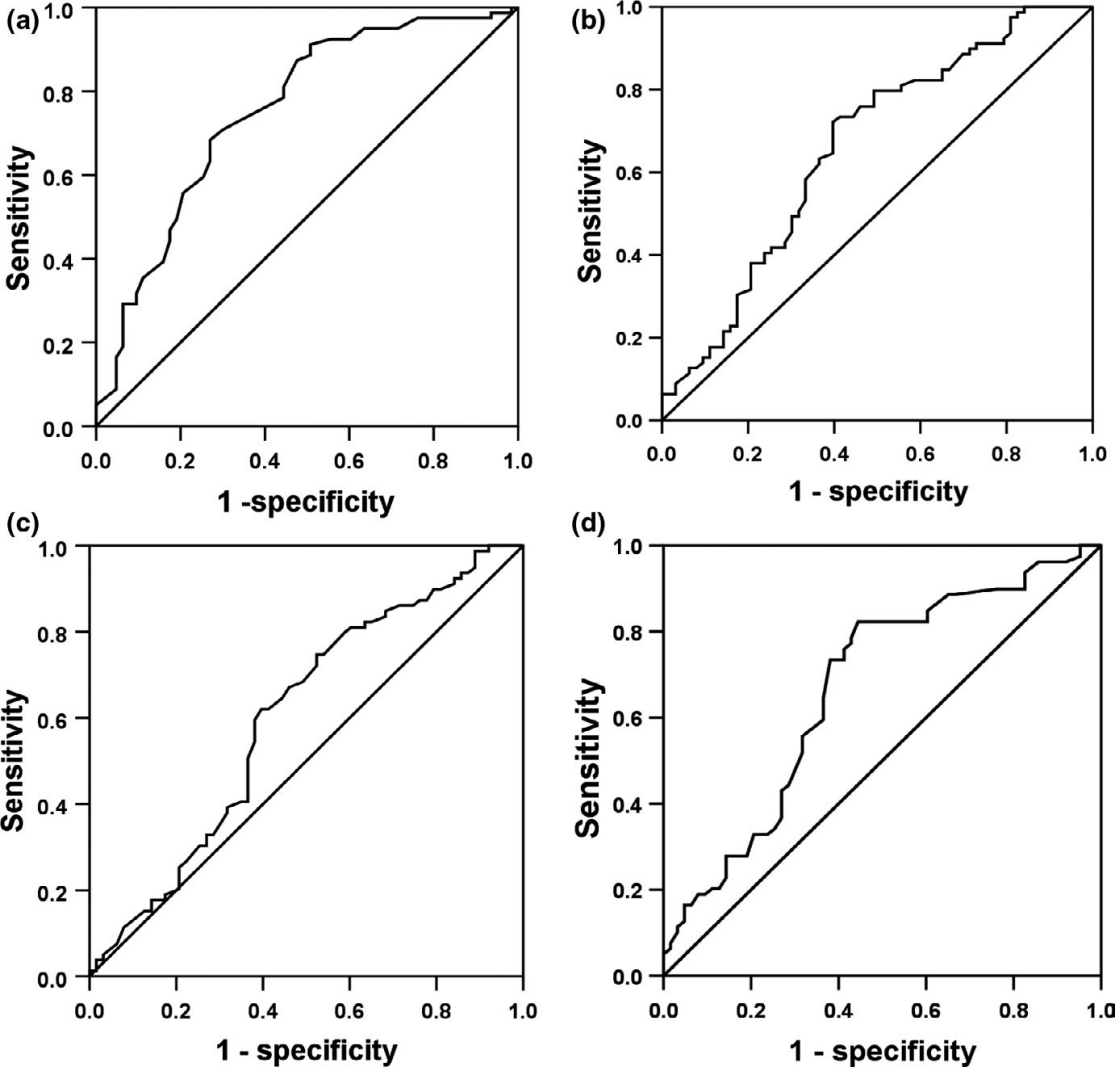
The receiver operating characteristic (ROC) curve of predictive value on POTS diagnosis between the supine and orthostatic electrocardiogram (ECG) index. (a) The ROC curve of the HR difference on POTS diagnosis. (b) The ROC curve of the T‐wave amplitude difference in lead V_4_ on POTS diagnosis. (c) The ROC curve of the T‐wave amplitude difference in lead V_5_ on POTS diagnosis. (d) The ROC curve of the T‐wave amplitude difference in lead V_6_ on POTS diagnosis. The y‐axis represents sensitivity, and the x‐axis represents the false‐positive rate (1‐specificity). The 45° straight line stands for the reference line indicating the sensitivity, and the false‐positive rate is equal

Draw ROC curve to evaluate the value of the HR difference and T‐wave amplitude difference in leads V_4_, V_5,_ and V_6_ on POTS diagnosis (Figure 2). The area under ROC curve of the HR difference was 0.744, showing moderate test efficiency. The area under ROC curve of the T‐wave amplitude difference in lead V_4_, V_5,_ and V_6_ was 0.667, 0.611, and 0.672, respectively, indicating low test efficiency. The sensitivity and specificity of POTS diagnosis were analyzed and predicted with different values as the boundary points, and we found that the HR difference was 15 times/min (sensitivity 69.0% and specificity 69.0%), the T‐wave amplitude difference in lead V_4_ was 0.10 mV (sensitivity 74.0% and specificity 60.6%), the T‐wave amplitude difference in lead V_5_ was 0.17 mV (sensitivity 59.0% and specificity 62.0%), and the T‐wave amplitude difference in lead V_6_ was 0.07 mV (sensitivity 80.0% and specificity 53.5%) as the optimal cutoff point.

### 3.4 Evaluate the cutoff point

We found when the HR difference was ≥15 times/min, the T‐wave amplitude difference in lead V_4_ was ≥0.10 mV, in lead V_5_ was ≥0.17 mV and in lead V_6_ was ≥0.07 mV simultaneously; the sensitivity, specificity, positive likelihood ratio, negative likelihood ratio and Youden index of POTS diagnosis in children and adolescents were 39.0%, 88.7%, 3.45, 0.69, and 27.7%, respectively. For the convenience of clinical application, we assigned the HR difference was ≥5 times/min, the T‐wave amplitude difference in lead V_5_ was ≥0.15 mV, and in lead V_4_ and V_6_ were ≥0.10 mV. The sensitivity, specificity, positive likelihood ratio, negative likelihood ratio and Youden index of POTS diagnosis were 35.0%, 88.7%, 3.10, 0.73, and 23.7%, respectively.

### 3.5 Follow‐up results

**Figure 3:**
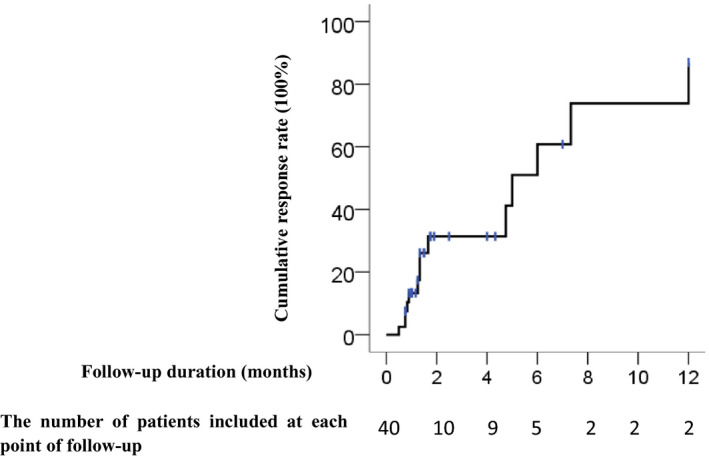
Cumulative response rate of POTS patients receiving treatment during follow‐up

**Figure 4:**
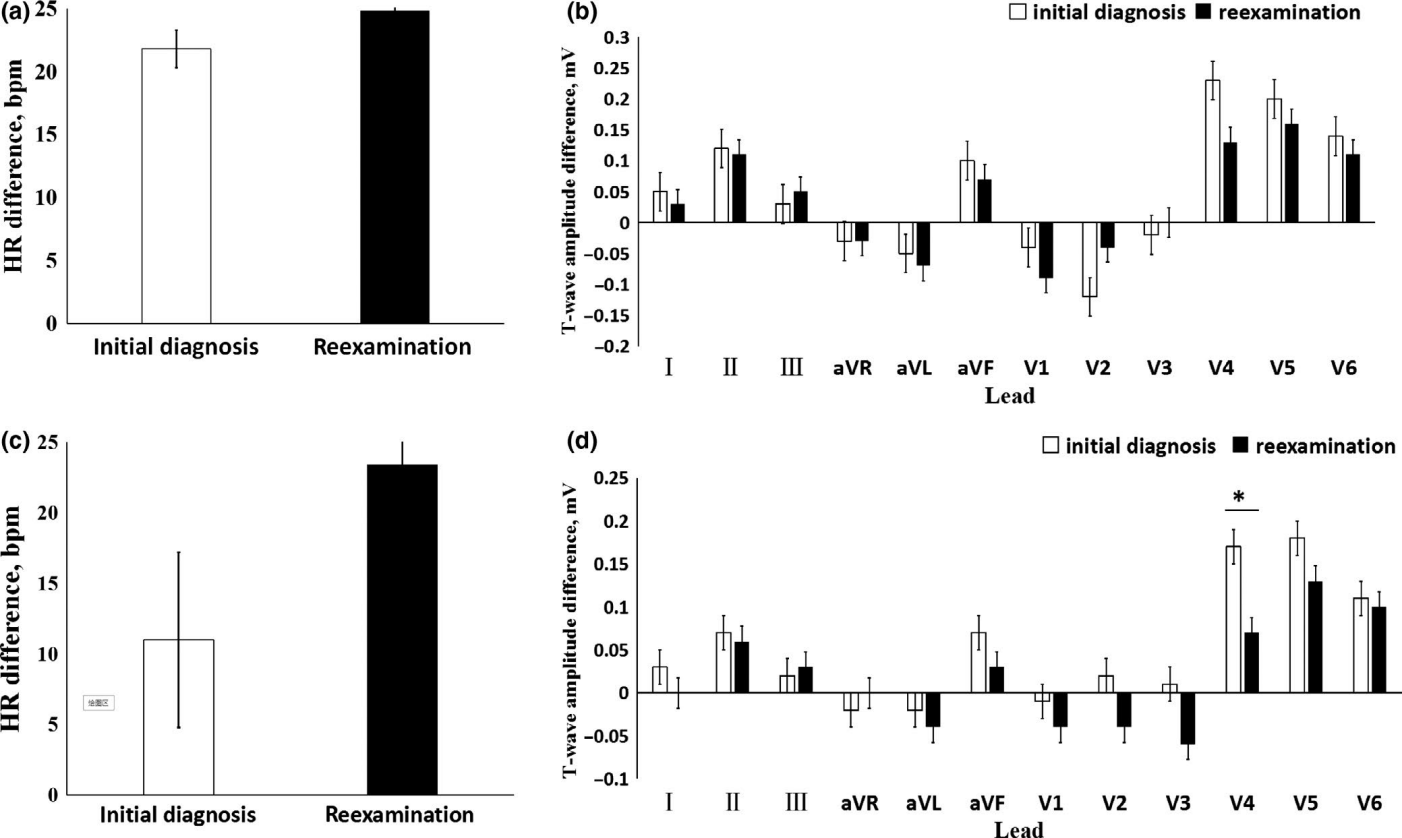
The reexamined HR difference and T‐wave amplitude difference on supine and orthostatic electrocardiogram compared with those of the initial diagnosis. (a) The reexamined and the initial HR difference in the nonresponsive group. (b) The reexamined and the initial T‐wave amplitude difference in the nonresponsive group. (c) The reexamined and the initial HR difference in the responsive group. (d) The reexamined and the initial T‐wave amplitude difference in the responsive group. * *p* < .05

All children and adolescents with POTS showed improved on symptoms of syncope and dizziness after treatment, and the increased HR was significantly lower than that before treatment (31.2 ± 13.9 times/min vs. 45.1 ± 14.2 times/min, *t* = 4.780, *p* < .01), among which 62% (26/40 cases) showed a response. The number of patients having a response was gradually increased over time. The cumulative response rates at 1, 2, 6, and 12 months after the beginning of follow‐up were 13.3%, 31.4%, 60.8%, and 86.9%, respectively (Figure 3). Children and adolescents were divided into groups according to the therapeutic effect. After reexamination of the supine and orthostatic ECG and comparison of the HR and T‐wave amplitude with the initial diagnosis value, no significant change was found in the nonresponsive group (*p* > .05). The T‐wave amplitude difference in lead V_4_ in the response group was decreased compared with that at the initial diagnosis (*p* < .05). There was no significant change of HR difference (*p* > .05) (Figure 4).

## 4 DISCUSSION

The main pathogenesis of POTS is hypovolemia, autonomic nervous dysfunction, hyperadrenergic, muscle pump dysfunction, and vascular endothelial dysfunction. These mechanisms can appear to co‐exist (Raj, 2013; Sheldon et al., 2015; Thanavaro & Thanavaro, 2011). Approximately 70% of POTS patients have hypovolemia, which is 15% lower than the healthy people, mainly related to the abnormal regional regulation of abdominal and lower limb blood vessels after standing (Fu et al., 2010). Decreased blood volume can stimulate the pressure receptors and cardiopulmonary receptors in the carotid sinus and aortic arch, so that the cardiac sympathetic nerves occupy a dominant position, resulting in increased HR. The increase of plasma adrenalin level in POTS patients can lead to that the vagus nerve be dominant, represented symptoms such as hyperhiddenness and cooling of extremities (Carew et al., 2009; Kanjwal, Saeed, Karabin, Kanjwal, & Grubb, 2011). Peltier et al. (2010) reported that the female POTS patients (17/30 cases) had abnormal axonal reflex on sweat gland regulating nerve of lower limb, suggesting that autonomic nerve in POTS patients is damaged. Li et al. (2014 found the existence of adrenoceptor antibodies in serum of POTS patients (*n* = 14), which can lead to compensatory activation of α1 receptor and β receptor in peripheral blood vessels, causing vasoconstriction and tachycardia, suggesting that autonomic nerve dysfunction in POTS patients is correlated with autoimmunity. To sum up, hypovolemia and autonomic nervous dysfunction of POTS patients can affect autonomic nervous regulation.

Autonomic nerve is closely related to cardiovascular system, and the interaction between sympathetic nerve and vagus nerve can reflect on ECG waveform. When the sympathetic nerve is dominant, HR increases, P‐wave amplitude increases, PR interval shortens, QRS duration shortens, T wave is low or inverted, and ST segment moves down. When the vagus nerve is dominant, HR slows down, P‐wave amplitude decreases, PR interval prolongs, QRS wave duration widens, T wave with high peaks, and ST segment lifts. T wave of ECG reflects 3‐phase repolarization of ventricular action potential, and its main ion current is K^+^ outflow (Xie, 2002). Changes in cardiac autonomic nervous tension and hypokalemia can cause changes in K^+^ channel permeability, thus affecting 3‐phase repolarization of action potential. Autonomic nerve dysfunction is the most common influencing factor, and 20% ~ 40% of T‐wave changes are related to it (Liu, 2006). Yeragani et al. (2000 reported that QT variation index was significantly lower in orthostatic and isoproterenol infusion than in supine position, suggesting that QT interval changes caused by postural changes are related to increased sympathetic activity. Sympathetic neurotransmitter isoproterenol can inhibit slowly activating delayed rectifier potassium current to slow K^+^ outflow and prolong 3‐phase repolarization. The innervation density of sympathetic epicardial region is greater than that of ventricular endocardial region, and its uneven distribution makes the repolarization speed of epicardial slow down more obviously, resulting in the decrease of T‐wave amplitude.

Lu et al. (2016 reported that compared with healthy children, QT interval dispersion and corrected QT interval dispersion of POTS children increased, but minimum QT interval and minimum corrected QT interval shortened. Baumert et al. (2011 research showed that compared with healthy children, the amplitude of T wave in children with POTS decreased more obviously during HUTT, but the change of QT interval was not obvious. Mayuga & Fouad‐Tarazi (2007 showed that T‐wave changes during HUTT were associated with POTS and vasovagal syncope, among which T‐wave changes in leads II, III, aVF, and V_3_ ~ V_6_ were correlated with POTS. Li et al. (2013 reported that in 122 children aged 6–13 years old who complained of chest tightness of unknown cause and/or chest pain in resting period, the difference of T‐wave amplitude and HR between the spine and orthostatic ECG in the HUTT positive group (*n* = 61) was increased compared with the HUTT negative group (*n* = 61) in leads Ⅱ, Ⅲ, aVF, and V_5_ (*p* < .05). Our results showed that the HR in orthostatic position was higher than that in supine position (*p* < .01), and the T‐wave amplitude in leads Ⅰ, Ⅱ, Ⅲ, aVF, V_4_, V_5,_ and V_6_ decreased (*p* < .05) in POTS group, which was basically consistent with the above results. Compared with the control group, the HR difference increased and the T‐wave amplitude difference in leads I, II, aVL, V_4_, V_5,_ and V_6_ increased in POTS group. Logistic regression analysis showed that the HR difference and the T‐wave amplitude difference in leads V_4_, V_5,_ and V_6_ between supine and orthostatic ECG had statistical significance for POTS diagnosis. When the HR difference was ≥15 times/min, the T‐wave amplitude difference in lead V_5_ was ≥0.15 mV, T‐wave amplitude difference in lead V_4_ and V_6_ were ≥0.10 mV, and the sensitivity and specificity for POTS diagnosis were 35.0% and 88.7%, respectively.

During the follow‐up of children and adolescents with POTS, there was no significant difference in the HR difference and the T‐wave amplitude difference between the reexamination and the initial diagnosis value of the nonresponsive group. For the responsive group, the T‐wave amplitude difference in lead V_4_ was lower than the initial value, but there was no significant difference in HR difference. It is suggested that supine ECG and orthostatic ECG have no significance for follow‐up of children and adolescents with POTS.

## 5 LIMITATION

It should be noted that few children completed the follow‐up in this study; therefore, the sample size needs to be expanded. Furthermore, the parameters showed low sensitivity. It may be related to insufficient standing time before tracing orthostatic ECG, which did not cause significant autonomic nervous system changes, or short maintenance time of ECG changes caused by autonomic nervous dysfunction (Ran et al., 2015). In addition, patients did not complete symptom scoring at initial diagnosis and follow‐up.

## 6 CONCLUSIONS

This study suggested that the HR difference and T‐wave amplitude difference in leads V4, V5, and V6 between supine and orthostatic ECG are of help in assisting the diagnosis of POTS but no obviously significance on prognosis estimation of it.

## CONFLICT OF INTEREST

The authors declare that they have no conflicts of interest.

## AUTHOR CONTRIBUTIONS

Yuwen Wang and Cheng Wang conceptualized and designed the study, performed the data analysis, interpreted the study findings, drafted the initial manuscript and revised the manuscript. Fang Li, Ping Lin, and Yuwen Wang collected the data. Yi Xu, Juan Zhang and Runmei Zou reviewed and revised the manuscript. All authors approved the final manuscript as submitted and agree to be accountable for all aspects of work.

## ETHICS

The study was approved by the Ethics Committee of the Second Xiangya Hospital of Central South University. All patients provided written informed consent.
